# HPV Knowledge Among a Marginalized Population [Letter]

**DOI:** 10.5888/pcd10.130088

**Published:** 2013-03-28

To the editor:

The original research article by Mills et al (1) provides important insight into the barriers for human papillomavirus (HPV) vaccine uptake among a marginalized population. Our group also examined obstacles to HPV vaccination among an underserved population – Latinas. This population is similar to the Appalachian population with respect to cervical cancer incidence, mortality, and HPV vaccination uptake. Incidence and death rates for Latinas are 50% to 70% higher than for non-Hispanic white women (2). There is also significant disparity in vaccine uptake between Latinas and non-Hispanic whites (3,4). Explanations for these findings include poor access to health care, lack of knowledge or health literacy, immigration status, and cultural beliefs (5,6).

Unpublished data from our group suggest that lack of knowledge about HPV may play a crucial role in shaping Latinas’ use of cervical cancer preventive health service. Using a convenience sample of Latina women recruited from 2 clinics serving indigent populations in 2009, we performed descriptive analyses of an HPV knowledge survey. The number of correct responses to 5 questions acted as a measure of knowledge.

A total of 86 Latina women participated: 80% were of Mexican origin. Among the participants, 54% correctly identified HPV as the most common sexually transmitted infection (STI), 54% responded incorrectly that herpes was the most common STI, 74% correctly responded that HPV causes cancer, 29% correctly answered that HPV can cause penile cancer, and only 12% responded correctly that HPV may not have symptoms.

Chi-square analyses revealed that those who already had received the vaccine scored higher on the knowledge questions (*P* = .005). In addition, scores were higher for those who feel they speak English better than Spanish (*P* = .06), those who read or speak primarily in English (*P* = .03), those who think primarily in English (*P* = .04), and those who speak in English with friends (*P* = .05).

Further examination found that those who speak or read primarily in English (*P* = .005), those who think in English (*P* = .04), those who feel they speak English best (*P* = .008), and those that speak in English with friends (*P* = .03) were more likely to have received the vaccine.

Most of this sample of underserved Latinas correctly responded that HPV is associated with cervical cancer. The more knowledgeable were the more vaccinated. However, the highest level of knowledge was associated with those who are comfortable with the English language.

Our findings echo what was found with young Appalachian women: there is a knowledge gap among HPV-unvaccinated women. We agree that educational interventions for at-risk populations are needed. However, many distinct populations are vulnerable to HPV and cervical cancer, and the complexity and cost of customizing a campaign for each group may prove too expensive. The question is how to effectively reach these populations in a cost-effective manner, transcending the differences among them. Lay health workers, peers, and others who are trusted within the community could offer education about cervical cancer, HPV, and prevention. Pharmacies in neighborhoods with high proportions of vulnerable women could act as nontraditional HPV vaccination delivery sites, overcoming barriers such as limited free time and difficulty with transportation. Pediatricians’ offices could be universally equipped to offer HPV vaccines to mothers. Beyond applying some of the population-specific strategies mentioned by Mills et al, we recommend universal implementation of aggressive, unconventional methods to decrease disparities in vaccine uptake and the disproportionately large burden of cervical cancer faced by underserved women nationwide.

**Anna M. Acosta, MD **Emory University School of Medicine 49 Jesse Hill Jr Dr SE  Atlanta, Georgia 30333 

**Loida E. Bonney, MD **Emory University School of Medicine 49 Jesse Hill Jr Dr SE Atlanta, Georgia 30333 

**Michael Fost, MSc **Emory University School of Medicine 49 Jesse Hill Jr Dr SE Atlanta, Georgia 30333 

**Victoria L. Green, MD **Emory University School of Medicine 49 Jesse Hill Jr Dr SE Atlanta, Georgia 30333 

**Carlos del Rio, MD **Emory University School of Medicine and Rollins School of Public Health of Emory University 49 Jesse Hill Jr Dr SE Atlanta, Georgia 30333 
